# Items, Selections and Remarks

**Published:** 1870-10

**Authors:** W. W. Miner


### Items, Selections and Remarks.

BY W. W. MINER, A. B.

In San Francisco, recently, the sudden and unaccountable death of a boy ten years of age, led at length to the arrest of his father on suspicion, when, by chance, the physician who conducted the autopsy, after its completion bethought himself to examine and remove the larynx, this organ was found to contain a large mass of meat, which produced death by suffocation. It appeared that the boy had in the night been awakened by an attack of vomiting, during which the meat became lodged in its position. The *Pacific Medical*

*Journal* gives an account of another case also, in which a patient who was put under the influence of chloroform, after having eaten a hearty meal, during an attack of vomiting lodged a portion of food in his larynx and was suffocated in spite of every effort.——One of the French journals says that a society has been formed in Paris, now numbering more than a hundred members, each of whom declare that it is their wish that their bodies, after death, be used for the promotion of anatomical science.

Division of structures, by means of the galvano-caustic wire, seems to be in vogue at present.——M. Baikow states that in set of experiments, twenty-eight in number, he has been successful in fourteen of them in producing bony substance by the insertion of marrow from a dog's femur, beneath the skin of his back, a method upon which M. Gonjon has heretofore experimented.——Prof. John T. Darby, of the University of South Carolina, has published a pamphlet on the use of liquid glass as a surgical dressing for fractures. He says that after an extensive use of plaster, starch, glue and dextrine, he is induced to place this substance above these for general usefulness. Six cases in which he made use of the soluble glass are given, which, together with the opinion of a number of surgeons, testify as to its superior utility.

The Wisconsin State Medical Society held its annual meeting at Milwaukee in June. Dr. H. P. Strong, of Beloit, was elected President of the Society for the ensuing year, and Dr. J. T. Reeve, of Appleton, Secretary.——The American Pharmaceutical Association met last month in Baltimore, holding a three days' session. Its next meeting will be held in St. Louis, in September, 1871. Its present officers are Richard H. Stubbs, of Virginia, President; John M. Maisch, of Philadelphia, Secretary.

M. Mehu, Pharmacist of the Becker Hospital, Paris, recommends, after several years' trial and experimenting, the following preservative fluid for anatomical specimens. It is especially valuable in not contracting the soft parts: Arsenious acid, 20 parts; crystalized carbolic acid, 10 parts; alcohol, 300 parts; distilled water 700 parts.—*N. Y. Medical Journal.*

In the *Dental Register*, for September, may be found an address delivered before the New York State Dental Society at its last annual meeting, by its estimable President, Dr. B. T. Whitney, of this city. From the address we learn that James Gardette, who came from France to America with a naval detachment of Lafayette's army in 1788, was the first to substitute gold clasps for ligatures of wire or thread in sustaining artificial teeth; thus enabling the patient to remove and cleanse them without the aid of a dentist. He was the first to support an artificial set of teeth by atmospheric pressure, which he did as early as the year 1800; artificial dentures were then made by carving them from blocks of ivory or the tooth of the hippopotamus. He was also the first to use gold plates as a base for teeth, in place of the clumsy carved pieces of perishable material, and was among the first to use gold foil in place of the lead and tin so generally used at that time.

